# ﻿Description of *Ixodeslanigeri* sp. nov., a new hard tick species (Acari, Ixodidae) collected from mouse-eared bats (Vespertilionidae, *Myotis*) in Vietnam

**DOI:** 10.3897/zookeys.1215.123624

**Published:** 2024-10-14

**Authors:** Sándor Hornok, Jenő Kontschán, Ai Takano, Yasuhiro Gotoh, Alexandre Hassanin, Vuong Tan Tu

**Affiliations:** 1 Department of Parasitology and Zoology, University of Veterinary Medicine, Budapest, Hungary University of Veterinary Medicine Budapest Hungary; 2 HUN-REN-UVMB Climate Change: New Blood-sucking Parasites and Vector-borne Pathogens Research Group, Budapest, Hungary HUN-REN-UVMB Climate Change: New Blood-sucking Parasites and Vector-borne Pathogens Research Group Budapest Hungary; 3 Plant Protection Institute, HUN-REN Centre for Agricultural Research, Budapest, Hungary Plant Protection Institute, HUN-REN Centre for Agricultural Research Budapest Hungary; 4 Department of Plant Sciences, Albert Kázmér Faculty of Mosonmagyaróvár, Széchenyi István University, Mosonmagyaróvár, Hungary Széchenyi István University Mosonmagyaróvár Hungary; 5 Department of Veterinary Medicine, Joint Faculty of Veterinary Medicine and The United Graduate School of Veterinary Science, Yamaguchi University, Yamaguchi, Japan Yamaguchi University Yamaguchi Japan; 6 Department of Bacteriology, Faculty of Medical Sciences, Kyushu University, Fukuoka, Japan Kyushu University Fukuoka Japan; 7 Institut Systématique Evolution Biodiversité, Sorbonne Université, Muséum national d’Histoire naturelle, Paris, France Sorbonne Université Paris France; 8 Institute of Ecology and Biological Resources, Vietnam Academy of Science and Technology, Hanoi, Vietnam Vietnam Academy of Science and Technology Hanoi Vietnam; 9 Graduate University of Science and Technology, Vietnam Academy of Science and Technology, Hanoi, Vietnam Graduate University of Science and Technology Hanoi Vietnam

**Keywords:** Chiroptera, *Myotisalticraniatus*, *Myotislaniger*, new species, *Pholeoixodes*, Southeast Asia, taxonomy

## Abstract

Historically, for more than one and a half centuries, only one so-called “long-legged bat tick” species, i.e., *Ixodesvespertilionis* Koch was known to science. However, during the past decade, it was recognized on a molecular basis that long-legged ixodid ticks associated with bats may represent at least six species. Of these, until recently, five have been morphologically described. In this study, *Ixodes* ticks were collected from two *Myotis* species in southeastern Asia, Vietnam. Based on the morphological and molecular characteristics of the female, nymph and larva, *Ixodeslanigeri* Hornok, **sp. nov.** is described here. The male is unknown. Like other members of the *Ixodesariadnae* complex, *I.lanigeri* Hornok apparently shows a preference for vesper bats as its typical hosts. In this context, host-association and geographical separation may explain the evolutionary divergence of *I.lanigeri* Hornok from its closest relative occurring on *Murinahilgendorfi* Peters in East Asia, Japan, because no *Myotis* or *Murina* spp. have overlapping distribution between Vietnam and the main islands of Japan. On the other hand, supposing that (similarly to *I.ariadnae*) *I.lanigeri* Hornok probably occurs on other myotine bats and knowing that several *Myotis* species indigenous in Vietnam have a broad geographical range in southern and southeastern Asia, the new tick species most likely has a widespread distribution in this area.

## ﻿Introduction

Hard ticks (Acari: Ixodidae) are haematophagous arthropods that affect their vertebrate hosts in multiple ways, causing skin lesions, blood loss, and, most importantly, transmitting tick-borne pathogens (Jongejan and Uilenberg 2004). Although ixodid ticks (as arachnids in general) cannot fly, their association with flying vertebrate hosts, such as birds and bats, make them capable of travelling large geographical distances (de la Fuente et al. 2015).

In the context of Eurasia, latitudinal (west-to-east/southeast) connectedness of tick populations via flying vertebrate hosts shows significant differences depending on whether they associate with birds or bats. Bird migration allows unrestricted gene flow, as reflected by the near genetic identity of conspecific hard ticks collected from avian hosts in Central Europe and the Far East, Japan (Hornok et al. 2016a). On the other hand, while a similar phenomenon was observed among bat soft ticks (Acari: Argasidae) between Central Europe and Central Asia (Hornok et al. 2017a), populations of taxonomically closely related bat-associated hard ticks (Acari: Ixodidae) in Europe and Southeast Asia show remarkable morphological and genetic differences and appear to be reproductively isolated. This can explain why long-legged ixodid ticks collected from horseshoe bats (Rhinolophidae) in Europe and Southeast Asia (Vietnam) were recognized to belong to different species, from the latter region described under the name *Ixodescollaris* Hornok, 2016 (Hornok et al. 2016b).

Most recently, the detailed morphological and genetic analyses of ixodid ticks collected from bats in Japan (i.e., in the Far East) revealed that these are different from those in Europe and deserve taxonomic status as separate species (Takano et al. 2023). While one of the two long-legged bat tick species discovered in Japan (*Ixodesnipponrhinolophi* Hornok & Takano, 2023: a member of the *I.vespertilionis* complex) was also shown to be different from its sibling species in Southeast Asia (*I.collaris*, occurring in Vietnam), the second long-legged species (*Ixodesfujitai* Hornok & Takano, 2023: member of the *Ixodesariadnae* Hornok, 2014 complex) was not compared in a similar context. The typical hosts of the latter group of ticks are vesper bats (Vespertilionidae), most notably *Myotis* and *Murina* species. The aim of this study was to compensate for the lack of data on members of the *I.ariadnae* complex in Vietnam, i.e., to characterize long-legged bat ticks collected from *Myotis* spp. in this country with both morphological and molecular biological methods.

## ﻿Material and methods

### ﻿Sample collection

**Figure 1.**
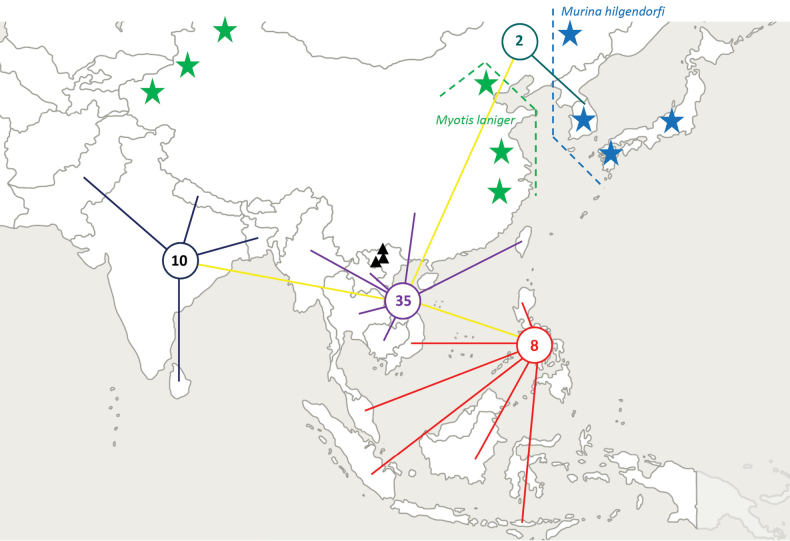
Map of Southeast and East Asia showing the number of *Myotis* and Murininae species indigenous in Vietnam and also occurring (1) in four countries (connected with dark blue lines) of the Himalayan and Indian subregions (*N* = 10 bat species), (2) in six countries (connected with purple lines) of the Indochinese (*N* = 35 bat species) subregion, as well as (3) in six countries (connected with red lines) of the Sundaic, Wallacean and Philippine (*N* = 8 bat species) subregions and (4) only two *Myotis* species from Vietnam occurring in and near the Korean Peninsula (blue line). For further details and references see Suppl. material 1. Black triangles show the collection sites of type material in northern Vietnam; green stars mark the eastern and western distribution limits of *Myotislaniger*; blue stars show the southernmost range of *Murinahilgendorfi*. The list of the species according to the above four categories: (1) *My.altarium* Thomas, 1911; *My.annectans* (Dobson, 1871); *My.hasseltii* (Temminck, 1840); *My.laniger* (Peter, 1871); *My.muricola* (Gray, 1846); *My.formosus* (Hodgson, 1835); *Harpiocephalusharpia* (Temminck, 1840); *Mu.cyclotis* Dobson, 1872; *Mu.huttoni* (Peters, 1872); *Mu.leucogaster* Milne-Edwards, 1872. (2) *My.altarium* Thomas, 1911; *My.ancricola* Kruskop et al., 2018; *My.annatessae* Kruskop & Borisenko, 2013; *My.annamiticus* Kruskop & Tsytsulina, 2001; *My.annectans* (Dobson, 1871); *My.ater* (Peters, 1866); *My.chinensis* (Tomes, 1857); *My.hasseltii* (Temminck, 1840); *My.horsfieldii* (Temminck, 1840); *My.indochinensis* Son et al., 2013; *My.laniger* (Peters, 1871); *My.montivagus* (Dobson, 1874); *My.muricola* (Gray, 1846); *My.phanluongi* Borisenko et al. 2008; *My.pilosus* (Peters, 1869); *My.rosseti* (Oey, 1951); *My.siligorensis* (Horsfield, 1855); *My.formosus* (Hodgson, 1835); *My.rufoniger* Tomes 1858; *Harpiocephalusharpia* (Temminck, 1840); *Harpiolaisodon* Kuo et al., 2006; *Mu.annamitica* Francis & Eger, 2012; *Mu.beelzebub* Son et al., 2011; *Mu.chrysochaetes* Eger & Lim, 2011; *Mu.cyclotis* Dobson, 1872; *Mu.eleryi* Furey et al., 2009; *Mu.feae* (Thomas, 1891); *Mu.fionae* Francis & Eger, 2012; *Mu.harpioloides* Kruskop & Eger, 2008; *Mu.harrisoni* Crosba & Bates, 2005; *Mu.huttoni* (Peters, 1872); *Mu.kontumensis* Son et al., 2015; *Mu.leucogaster* Milne-Edwards, 1872; *Mu.lorelieae* Eger & Lim, 2011; *Mu.walstoni* Furey et al., 2011. (3) *My.ater* (Peters, 1866); *My.hasseltii* (Temminck, 1840); *My.horsfieldii* (Temminck, 1840); *My.muricola* (Gray, 1846); *My.siligorensis* (Horsfield, 1855); *Harpiocephalusharpia* (Temminck, 1840); *Mu.cyclotis* Dobson, 1872; *Mu.huttoni* (Peters, 1872). (4) *My.pilosus* (Peters, 1869); *My.rufoniger* Tomes 1858.

Ticks were removed from two species of mouse-eared bats (*Myotis* spp.) at three locations in northern Vietnam (Fig. 1). The type material is described below. After collection, the ticks were stored individually in vials containing 96% ethanol.

### ﻿Molecular-phylogenetic and morphological analyses

DNA was extracted from two larvae collected with and showing the same morphological characters as paratype #2. From one of these larvae, the complete mitogenome was amplified as reported (Takano et al. 2023). From the other larva, as well as from one leg of the female holotype and two legs of the nymph paratype#1, an approx. 710-bp-long part of the cytochrome *c* oxidase subunit I (*cox*1) gene and a 450-bp-long part of the 16S rRNA gene were amplified and sequenced as reported (Hornok et al. 2014). The latter sequences were used for phylogenetic analysis. Phylogenetic analysis was performed with MEGA 7 (Kumar et al. 2016), using 1000 bootstrap replicates and the Neighbor-Joining method, p-distance model. Measurements were performed and pictures were taken with a VHX-5000 digital microscope (Keyence Co., Osaka, Japan). The sizes in the descriptions below are provided in millimetres.

## ﻿Taxonomic account

**Family Ixodidae Koch**

**Genus *Ixodes* Latreille**

**Subgenus Pholeoixodes Schulze**

### ﻿ Ixodes lanigeri

Taxon classificationAnimaliaIxodidaIxodidae

Hornok sp. nov.

B852EA02-421D-561A-9914-8E628CA6784F

https://zoobank.org/F5F38340-44E4-45B0-8BFF-90C150548DD2

Figs 2 , 3 , 4 , 5

#### Diagnosis.

Medium size, light brown prostriate tick with drop shape body of the female. Legs long. Basis capituli dorsally pentagonal, palps short and hypostome medium length. Scutum reverse pentagonal, broadest at mid-length, posteriorly rounded, with long, deep and curved cervical grooves.

#### Material examined.

***Holotype***: • female from a female Himalayan whiskered bat (*Myotisalticraniatus* Osgood), collected in Vietnam (340 m a.s.l., Tho Than Cave, Xuan Son NP, Phu Tho Province: 21.138613°N, 104.939903°E) by Vuong Tan Tu on December 7, 2020. ***Paratype*** #1: • nymph from a male Chinese water myotis (*Myotislaniger* Peter), collected in Vietnam (1530 m a.s.l., Ta Phin # 1 Cave, Lao Cai Province: 22.402822°N, 103.836787°E) by Vuong Tan Tu on December 3, 2020. ***Paratype*** #2: • larva from a male Chinese water myotis (*M.laniger*), collected in Vietnam (1400 m a.s.l., Co Ma # 1 Cave, Co Ma Commune, Thuan Chau, Son La Province: 21.361139°N, 103.507718°E) by Vuong Tan Tu on December 17, 2020.

All above specimens are stored in ethanol and deposited at the Department of Parasitology, University of Veterinary Medicine, Budapest, Hungary (holotype and paratype #1) and the Institute of Ecology and Biological Resources, Hanoi, Vietnam (paratype #2).

#### Morphology.

**Figure 2.**
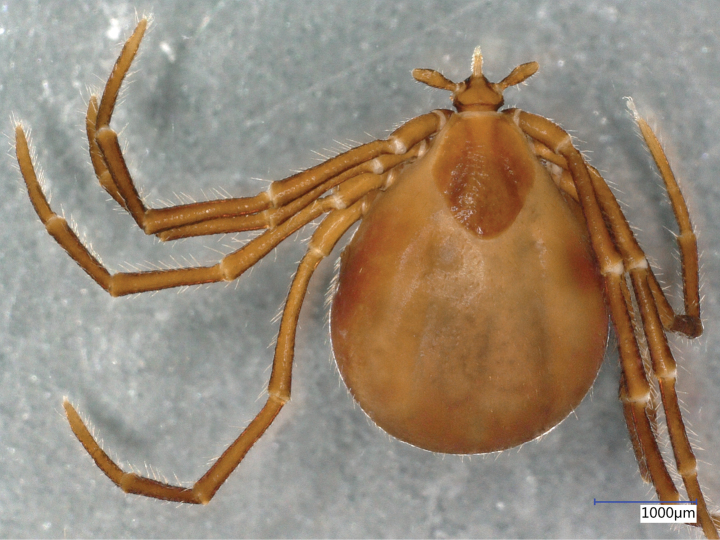
Dorsal habitus of *Ixodeslanigeri* sp. nov. female.

**Female (engorged)**. Length of the idiosoma (from the half point between scapular apices to the middle of posterior margin) 3.38, width 2.74, ratio of idiosomal length/width 1.23 (Fig. 2).

**Figure 3.**
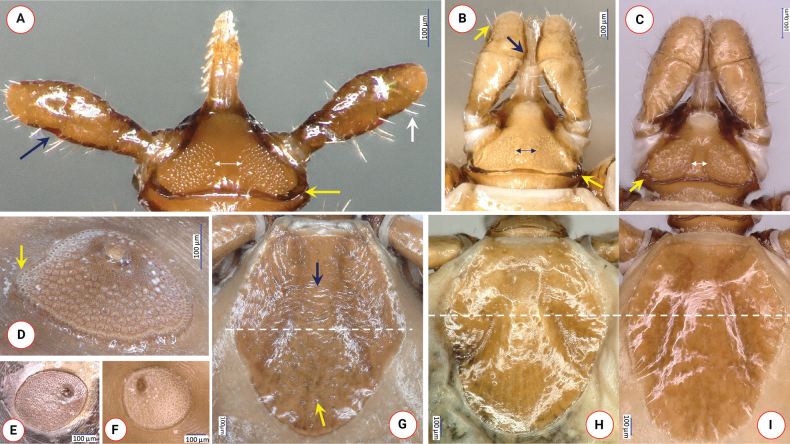
Key morphologic characters of *Ixodeslanigeri* sp. nov. female, in comparison with *I.fujitai* and *I.ariadnae***A–C** dorsal view of basis capituli of **A***I.lanigeri* sp. nov. (blue arrow: lateral protuberance of palpal segment II; white arrow: longest hair on palpal segment III; yellow arrow: caudolateral corner of basis; double white arrow: interval between porose areas) **B***I.fujitai* (blue arrow: medial protuberance of palpal segment III; white arrow: longest hair on palpal segment III; yellow arrow: caudolateral corner of basis; double blue arrow: interval between porose areas) and **C***I.ariadnae* (yellow arrow: caudolateral corner of basis; double white arrow: interval between porose areas) **D–F** Peritremes of **D***I.lanigeri* sp. nov. (yellow arrow: narrowing) **E***I.fujitai* and **F***I.ariadnae***G–I** scutum of **G***I.lanigeri* sp. nov. (yellow arrow: relatively dense punctuations, blue arrow: rugosities) **H***I.fujitai* and **I***I.ariadnae* (the dashed line indicates the maximum width of the scutum). Collection data of samples used for comparison: *I.fujitai* female was removed from *Murinahilgendorfi* in Shiga (Japan) on April 22, 2016; *I.ariadnae* female was collected from the wall of Legény Cave (Pilis Mountains, Hungary) on March 5, 2017.

Scutum reverse pentagonal, broadest at half-length, posteriorly rounded (Fig. 3G).

Length of scutum 1.26, maximum width 1.05, ratio length/width 1.2. On the scutum long, deep and curved cervical grooves, central and marginal rugosities and scattered punctuations visible (Fig. 3G). Caudolateral edge straight, with slight concavity where cervical grooves terminate. Scutal setae few, more evident laterally (length: 0.035).

Alloscutum with sparse hair covering dorsally. Length of centrodorsal setae 0.13, marginodorsal setae 0.1. Idiosoma with dense hair covering ventrally. Genital aperture flat W-shaped, with posterior concavity along its mid-line, situated slightly posterior to 2^nd^ intercoxal space. Genital groove diverging backwards, with concavity at the level of 4^th^ coxae. Spiracular plates asymmetrical, pear-shape, length 0.4, position of opening submarginal, surrounding aeropyles (around a gap of 0.06) in 2–7 rows (Fig. 3D). Anal valves with setae measuring 0.1. Anal groove slightly converging from mid-length.

**Figure 4.**
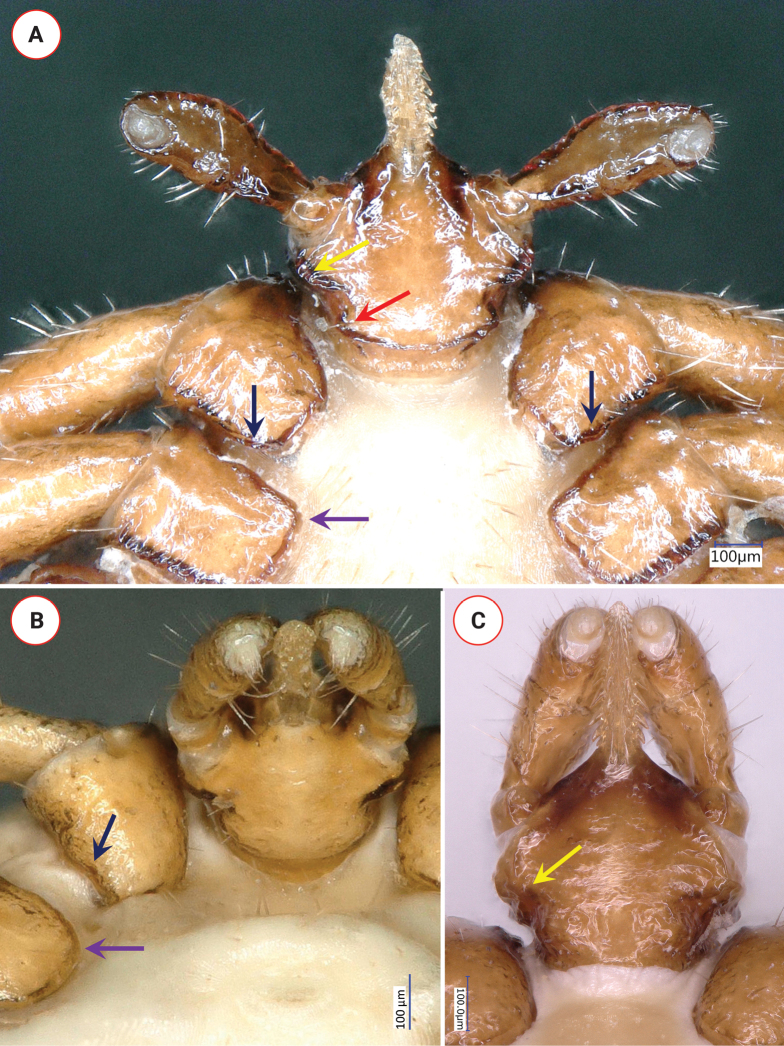
Key ventral morphological characters of *Ixodeslanigeri* sp. nov. female, in comparison with *I.fujitai* and *I.ariadnae***A** ventral view of *I.lanigeri* sp. nov. (blue arrow: rectangular coxa II; black arrow: sclerotized caudal protuberance of coxae I; yellow arrow: auriculae; red arrow: angled caudolateral corner of ventral basis) **B** ventral view of *I.fujitai* (blue arrow: rounded coxa II; black arrow: caudal concavity of coxa I) **C** ventral view of *I.ariadnae* (yellow arrow: auricular ridge).

Length of gnathosoma (from palpal apices to posterior margin of basis capituli) 0.6, width of basis capituli dorsally 0.5. Ratio of gnathosomal length to basis capituli width 1.2. Length of basis capituli (from base of hypostome to posterior margin of basis capituli) 0.33, ratio of length to width of basis capituli 0.66. Basis capituli shape pentagonal, its sides parallel, anteriorly converging (Fig. 3A). Caudolateral corner oblique, slightly rounded, without cornuae and continuing as a dark brown lane of sclerotization along the relatively straight posterior margin. Areae porosae very large, elliptical (with their longitudinal axes perpendicular to each other), their breadth 0.18, interval narrow (0.06). Ventrally on basis capituli prominent, caudolaterally projecting auriculae, bearing two longitudinal ridges, posteriorly tapering (Fig. 4A). Behind auriculae constriction (“waist”). Posterior edge of ventral basis medially less, laterally strongly sclerotized and caudolaterally angled (Fig. 4A), its width shorter than distance between palpal articles I, laterally with a single hair (0.03).

Palps (dorsal view) short, club shape, edge curved medially, relatively straight laterally, length 0.63, maximum width 0.22, ratio length/width 2.9. Palpal hairs few (i.e., 4–6) medially, more numerous (as many as 12–14) laterally, shortest (measuring 0.02) anteriorly, longest (measuring 0.08) posteriorly. Palpal segment I with slight anterior protuberance, ventrally with two setae (0.05) and longitudinal ridge. Palpal segment II 0.33 long, anteriorly broadening, with a strongly sclerotized longitudinal ridge ventrally, both medial and lateral concavity (fovea) near mid-length, and a lateral protuberance near the junction with segment III (Fig. 3A). Two caudolateral hairs of palpal segment II, in and near the lateral concavity, have medium length (0.05). Palpal segment III 0.26 long, laterally concave, medially convex (Fig. 3A). Hypostome slightly lanceolate, length 0.27, width 0.1, ratio length/width 2.7. Dental formula 2/2 (mid-length), in six rows (but apical part missing) (Fig. 4A).

Legs long, longer than 5 (Fig. 2). Coxae I asymmetrically trapezoid, coxae II rectangular, all coxae without spines or spurs but caudomedial angle of coxae I strongly sclerotized, with a slight protuberance laterally to it (Fig. 4A). A single coxal hair posterolaterally long (0.22), anterolaterally shorter (up to 0.1), except on coxae III where these two equal in length. Highest number of hair (*N* > 5) on coxae IV. Tarsus I. length 1.2, maximum diameter 0.1, length to diameter ratio 12. Haller’s organ open, with six anterior pit sensillae arranged as a group of three, and another three in line.

**Figure 5.**
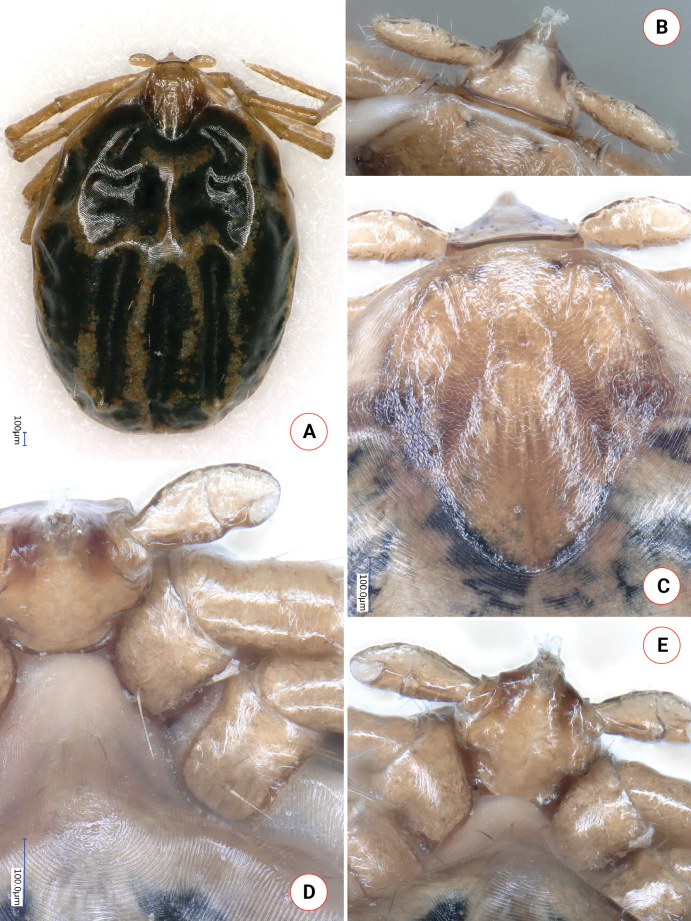
Key morphological characters of *Ixodeslanigeri* sp. nov. nymph **A** habitus, dorsal view **B** basis capituli **C** scutum and palps **D** coxae I-II **E** ventral view of basis and palps.

**Nymph (engorged)**. Length of the idiosoma 2.95 (Fig. 5A). Scutum broad, reverse pentagonal, broadest close to half-length (Fig. 5C). Length of scutum 0.61, maximum width 0.56, ratio length/width 1.1. On the scutum straight scapular groove measuring 0.1, and a relatively straight cervical groove reaching caudolateral margin at its middle, with a concavity. The surface has fine reticulate pattern. Punctuations not visible. Lateral scutal seta 0.04 long.

Alloscutum has few 0.08 long hair dorsally. Idiosoma has sparse hair covering ventrally (length: 0.04–0.05 between coxae, 0.1 in mid region and behind). Spiracular plates subcircular in shape, diameter 0.14, within marginal row scattered aeropyles in 1–3 rows, position of opening subcentral. Anal valves with four 0.06–0.07 long setae. Anal grooves nearly parallel.

Length of basis capituli (from base of hypostome to posterior margin of basis capituli) 0.13, width of basis capituli dorsally 0.22, ratio of length to width of basis capituli 0.6 (Fig. 5B). Basis capituli shape pentagonal, its sides slightly then (anterior to palpal basis) abruptly converging toward the hypostome, dorsally broadest at its caudolateral corners which are perpendicular, lacking cornuae. Posterior margin nearly straight. Three isolated pores observable in place of areae porosae. Ventrally on the basis triangular, sclerotized auriculae, with almost perpendicular lateral and caudal edges (Fig. 5E).

Palps (dorsal view) short, medial edge curved, lateral edge nearly straight (Fig. 5C), length 0.23, maximum width 0.095, ratio length/width 2.4. Palpal hairs longest (0.04) laterally on palpal segment II (*N* = 3) and slightly shorter (0.03) medially (*N* = 2). Palpal segment II and III 0.12 and 0.1 long, respectively (Fig. 5B). Palpal segment III narrower than palpal segment II at their junction, forming a laterally concavity. Palpal segment III with dorsal deepening (fovea), and laterally with five short (0.02) and anteriorly with shorter (0.01) hairs. Hypostome missing from paratype #2.

Legs long and slender. Coxae I trapezoid, their caudomedial corner perpendicular-angled, coxae II rectangular (Fig. 5D). Coxae II-IV rounded, without spines or spurs. Coxae I and II with long hair (0.11 and 0.09, respectively) medially at mid-length (Fig. 5D), all coxae with prominent hair of similar length (0.05–0.11) caudolaterally. Tarsus I. length 0.81, maximum diameter 0.09, length to diameter ratio 9.

**Figure 6.**
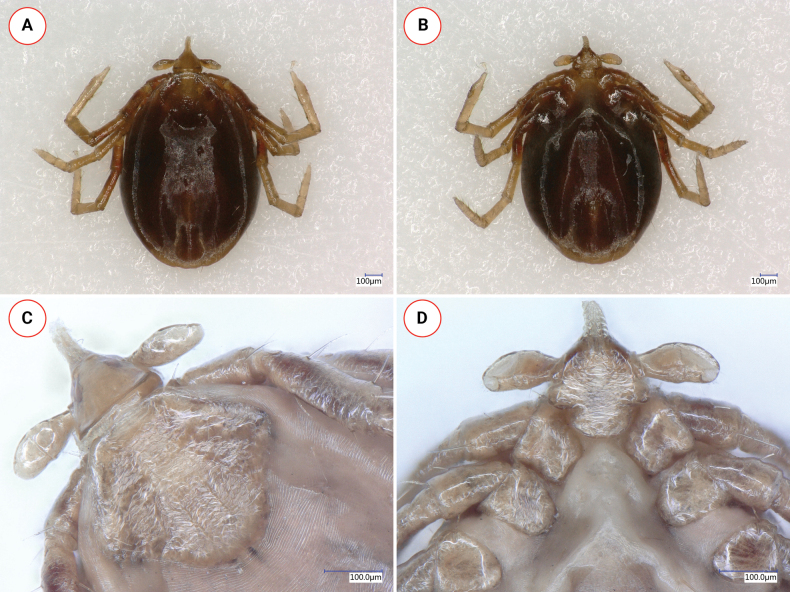
Key morphological characters of *Ixodeslanigeri* sp. nov. larva **A** habitus, dorsal view **B** habitus, ventral view **C** scutum, dorsal view of basis capituli and palps **D** coxae, ventral view of basis capituli and palps.

**Larva (engorged)**. Length of idiosoma 1.12, breadth 0.8, ratio idiosomal length/breadth 1.4 (Fig. 6A, B).

Scutum reverse pentagonal, posteriorly rounded, broadest at its half-length (Fig. 6C). Length of scutum 0.34, breadth 0.39, ratio length/breadth 0.87. Surface reticulate, with slight rugosities. Cervical grooves narrow, terminating close to deepest point of the pronounced concavity along curved caudolateral scutal margin (Fig. 6C). Between carinae and cervical grooves posterolaterally directed, anteriorly convex deepening. In the caudal field of scutum two parallel grooves with length of 0.08 and interval of 0.05 (Fig. 6C). Scutal setae few (Sc2: 0.024, Sc4: 0.036), some further dorsal and ventral setae also missing. Alloscutal setae longest around mid-length; central dorsal setae (Cd1-2: 0.05) shorter than marginal dorsal setae (Md1-3: 0.07, Md5: 0.08, Md6: 0.07, Md8: 0.04). Ventrally, sternal setae (St1: 0.033, St2: 0.044; St3: 0.07) mostly shorter than marginal ventral setae (Mv1: 0.067, Mv2: 0.086, Mv3: 0.073).

Gnathosoma: length from base of hypostome to posterior margin of basis 0.094, width of basis capituli dorsally 0.17, ratio of length to width 0.55. Basis capituli dorsally triangular, with straight posterior margin and rounded, oblique caudolateral corner, both showing a dark brown lane (<0.01) of sclerotization. Ventrally on the basis capituli elevated, blunt and triangular auriculae with sclerotized edge (Fig. 6D). Posterior margin rounded. Palps short, club-shaped, medially curved, laterally slightly convex with prominent dorsal fovea and lateral outward bulging of segment II near the junction with segment III (Fig. 6C). Dorsally, palpal length 0.15, breadth 0.07, ratio length/breadth 2.2. Segments I-III measure 0.01, 0.08 and 0.06, respectively. Palpal setae longest (0.015) apically and close to junction of II-III segments. Ventrally on palpal segment II porous elevation at the junction with segment III. Hypostome conical, short (0.11), with dental formula 2/2.

Legs long. Haller’s organ elongated, longer than maximum breadth (diameter) of tarsus I. Tarsus I length: 0.4, breadth: 0.058. Coxae without spines or spurs. Coxa I trapezoid, with caudomedial corner as elevated, perpendicular angle of dark sclerotization appearing as a short internal spur. Coxae II-III rounded.

#### Differential diagnosis.

*Ixodeslanigeri* sp. nov. can be distinguished from *I.simplex* Neumann and *I.fuliginosus* Hornok & Takano based on its long legs (tarsus I: length to maximum diameter ratio above 8), and from members of the *I.vespertilionis* complex (*I.vespertilionis*, *I.collaris*, *I.nipponrhinolophi*) based on its short palps, relevant to all known developmental stages.

Within the *I.ariadnae* complex, the female of *I.lanigeri* sp. nov. is different from *I.fujitai* based on the following characters of the latter: (1) scarce punctuations in the anterior and posterior fields of scutum (Fig. 3H) (*vs* denser in *I.lanigeri* sp. nov.); (2) subcircular spiracular plates (Fig. 3E) (*vs* asymmetrical, pear-shape in case of *I.lanigeri* sp. nov.); (3) gnathosoma approximately 30% longer than broad (*vs* only approximately 20% longer than broad in *I.lanigeri* sp. nov.); (4) angled, thickened and protruding caudolateral corners of basis capituli (Fig. 3B) (*vs* rounded and blunt, oblique in *I.lanigeri* sp. nov.); (5) subtriangular areae porosae with a broader interval of 0.08 (Fig. 3B), ratio of width-to-interval 2:1 (*vs* broad elliptical areae porosae, with their longitudinal axes perpendicular to each other and a narrower interval of 0.06 in *I.lanigeri* sp. nov., with a 3:1 ratio of width-to-interval); (6) lack of sagittal rim anteriorly on palpal article I (Fig. 4B) (*vs* observable in *I.lanigeri* sp. nov.); (7) lack of strongly sclerotized longitudinal ridge ventrally at the basis of palpal article II (Fig. 4B) (*vs* present in *I.lanigeri* sp. nov.), and the two caudolateral hairs of palpal segment II, in and near the lateral concavity, are long (0.1) in *I.fujitai* (Fig. 4B) (*vs* short, 0.05 in *I.lanigeri* sp. nov.); (8) slightly elevated and sclerotized auricular ridge (Fig. 3B) (*vs* prominent auriculae in *I.lanigeri* sp. nov.); (9) rounded posterior margin of ventral basis (Fig. 4B) (*vs* caudolaterally angled posterior margin of *I.lanigeri* sp. nov.); and (10) rounded coxae, especially coxae II (Fig. 4B) and IV (*vs* rectangular coxae II in *I.lanigeri* sp. nov.).

Differences in comparison with females of *I.ariadnae* include the following characters. In *I.ariadnae* the scutum is slightly more elongated (Fig. 3I) (ratio length/width above 1.25 *vs* 1.2 in *I.lanigeri* sp. nov.) and has its maximum width at approximately one-third of its length (*vs* close to half-length in *I.lanigeri* sp. nov.). The straight portion is in the middle of the cervical grooves in *I.ariadnae* (Fig. 3I), but posteriorly in *I.lanigeri* sp. nov. The number of pores is low in the caudal region of the scutum of *I.ariadnae* (Fig. 3I) *vs* higher in *I.lanigeri* sp. nov. The spiracular plates of *I.ariadnae* are subcircular (Fig. 3F), with straight portions of its edges (*vs* asymmetrically pear-shaped in *I.lanigeri* sp. nov.), diameter smaller (0.33 *vs* 0.4 in *I.lanigeri* sp. nov.). Aeropyles occupy up to 10 rows in *I.ariadnae* (*vs* up to 7 in *I.lanigeri* sp. nov.), with narrower margin than the diameter of their opening (*vs* broad margin in *I.lanigeri* sp. nov.). Gnathosoma approximately 30% longer than broad (*vs* only approximately 20% longer than broad in *I.lanigeri* sp. nov.). Dorsally, basis capituli with straight oblique caudolateral corner and wavy edge including that of posterior margin which is strongly concave in middle (Fig. 3C), *vs* rounded corner and straight posterior margin in *I.lanigeri* sp. nov. Shape of areae porosae subtriangular in *I.ariadnae* (Fig. 3C) *vs* elliptical in *I.lanigeri* sp. nov. Palps of *I.ariadnae* broader, with length-to-width ratio of 2.4 (Fig. 3C) (*vs* 2.9 in *I.lanigeri* sp. nov.). Ratio of palpal segments II:III 1.6 in *I.ariadnae* vs 1.27 in *I.lanigeri* sp. nov. Palpal segment II is laterally concave at its basis in *I.ariadnae* (Fig. 3C) (*vs* close to its mid-length both laterally and medially in *I.lanigeri* sp. nov.). Surface of palpal segment III convex both laterally and medially in *I.ariadnae* (Fig. 3C) (*vs* laterally concave, medially convex in *I.lanigeri* sp. nov.). Ventrally on the basis capituli of *I.ariadnae* “waist” (narrowing) and sclerotized posterior edge less evident, unangled and inconspicuous auricular ridges have convex, rounded anterior margin (Fig. 4C) (*vs* concave anterior margin surrounding palpal article I. in *I.lanigeri* sp. nov.). Coxae II-IV of *I.ariadnae* are symmetrically rounded *vs* asymmetrically trapezoid or rectangular in *I.lanigeri* sp. nov.

**Figure 7.**
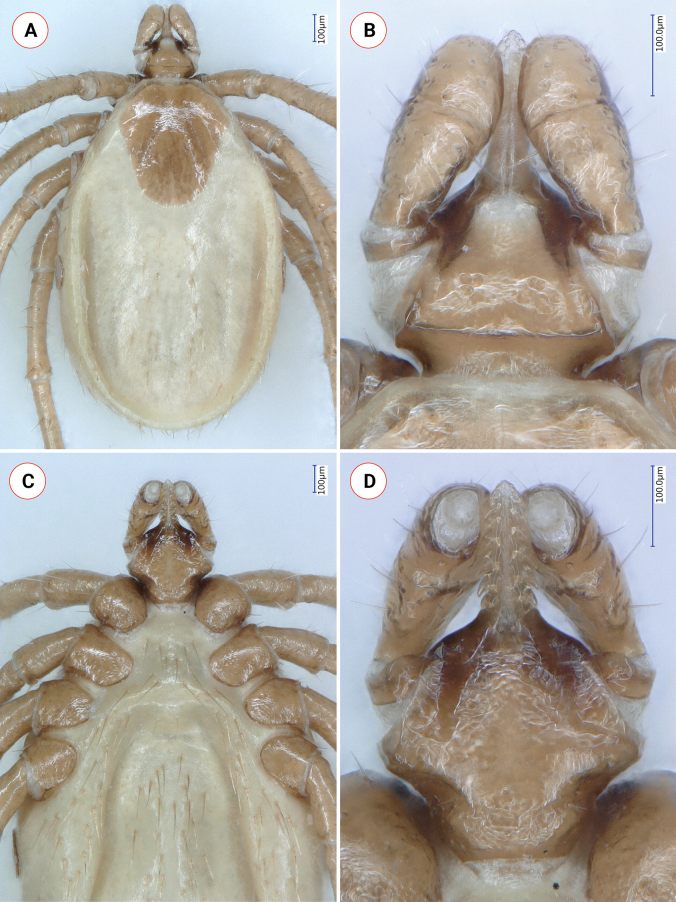
Key morphologic characters of *Ixodesariadnae* nymph **A** habitus, dorsal view **B** dorsal view of basis capituli and palps **C** coxae and ventral view of gnathosoma **D** ventral view of basis and palps. This sample was collected from the cave wall of Szopláki Ördöglyuk (Pilis Mountains, Hungary) on April 10, 2016.

In comparison with *I.collaris* nymph: palps slender, elongated, 0.45 (*vs* short, 0.23 in *I.lanigeri* sp. nov.) and the scutum is also more elongated (shape index is 1.5 *vs* 1.1 in *I.lanigeri* sp. nov.). Within the *I.ariadnae* complex, the nymph of *I.fujitai* is unknown. The nymph of *I.lanigeri* sp. nov. is different from that of *I.ariadnae* based on the following characters of the latter. The scutum is longer, as indicated by the shape index of 1.2, and broadest at its anterior third (Fig. 7A) (*vs* 1.1 in *I.lanigeri* sp. nov., maximum width close to half-length in *I.lanigeri* sp. nov.). The ratio of palpal segments II:III 1.7 (Fig. 7B) (*vs* 1.2 in *I.lanigeri* sp. nov.). Ventrally, on the basis of *I.ariadnae* nymph, less elevated auricular ridges are visible (Fig. 7D). Spiracular plates oval, with irregular outline in *I.ariadnae* nymph but subcircular in *I.lanigeri* sp. nov. Coxae of *I.ariadnae* nymph are all rounded (Fig. 7C).

**Figure 8.**
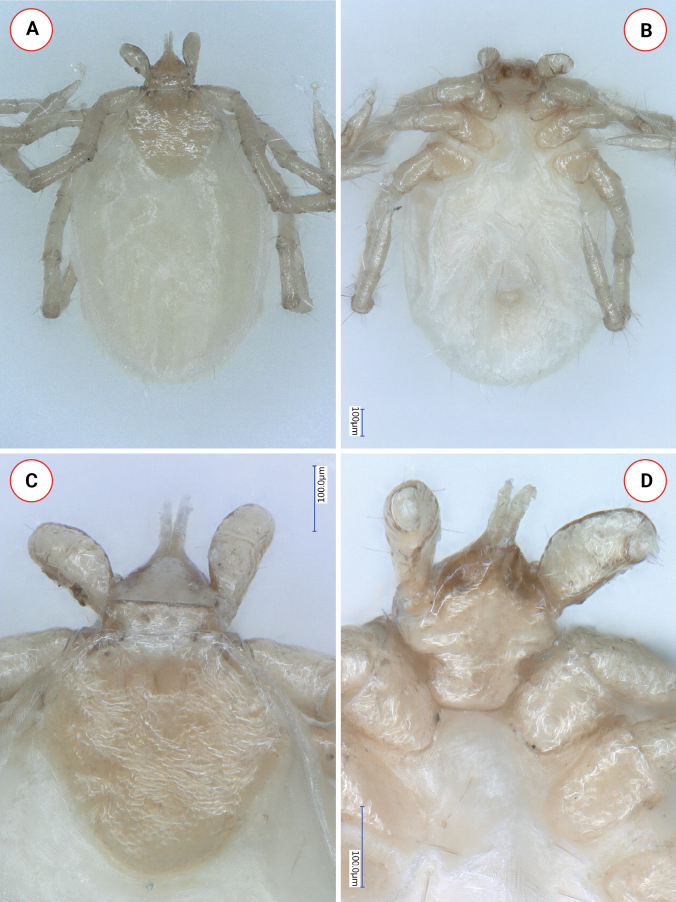
Key morphological characters of *Ixodesariadnae* larva **A** habitus, dorsal view **B** habitus, ventral view **C** scutum, dorsal view of basis capituli and palps **D** coxae I-II, ventral view of basis capituli and palps. This sample was collected from the cave wall of Szopláki Ördöglyuk (Pilis Mountains, Hungary) on April 10, 2016.

Within the *I.ariadnae* complex, the larva of *I.fujitai* is unknown. The larva of *I.lanigeri* sp. nov. is different from that of *I.ariadnae* (Fig. 8A, B) based on the following characters of the latter. Scutum broadest anteriorly to its half-length; its caudolateral edge with only slight concavity (Fig. 8C) (*vs* deep in *I.lanigeri* sp. nov). Cervical grooves not apparent, reaching posterolateral margin of scutum behind its deepest point of concavity. Scutal setae shorter (0.02–0.03) than in *I.lanigeri* sp. nov. (0.05). Caudal alloscutal setae longer in *I.ariadnae* larva (0.15 *vs* 0.04–0.08 in *I.lanigeri* sp. nov.). Marginal ventral setae longer in *I.ariadnae* larva (0.12–0.14 vs 0.06–0.09 in *I.lanigeri* sp. nov.). Palps of *I.ariadnae* larva laterally straight, with small fovea and dark outline (Fig. 8C) (*vs* laterally convex, with prominent fovea in *I.lanigeri* sp. nov.), but shape index is 2.2 in both species. Palpal setae longer in *I.ariadnae* (up to 0.05) than in *I.lanigeri* sp. nov. (0.015). Ventrally, on the basis, capituli auricular ridges of *I.ariadnae* are less apparent/elevated (Fig. 8D) than the auriculae in *I.lanigeri* sp. nov.

#### GenBank data.

Complete mitochondrial genome sequence from one larva is available in GenBank (LC797956). Sequences of the amplified parts of the *cox*1 and 16S rRNA genes of *I.lanigeri* sp. nov. from another larva (collected with paratype#2), the nymph (paratype#1) and the female (holotype) are found under the accession numbers PP079465, PP503326, PP503327 and PP081435, PP505539, PP505540 respectively.

#### Molecular and phylogenetic analyses.

**Figure 9.**
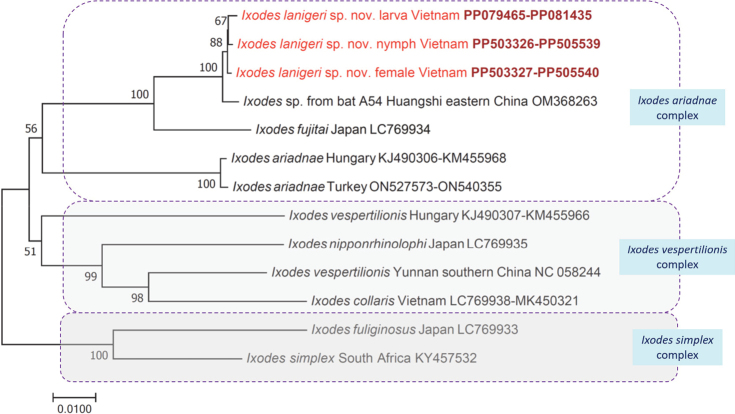
Phylogenetic tree of bat-associated ticks based on concatenated *cox*1 and 16S rRNA gene sequences. In each row of individual sequences, the region/country of origin and the GenBank accession number are shown after the species name. Rows of sequences from this study are indicated with red fonts and bold accession numbers. The evolutionary history was inferred by using the Neighbor-Joining method and p-distance model. The percentage of trees in which the associated taxa clustered together is shown next to the branches. Branch lengths are measured in the number of substitutions per site. The analysis involved 13 nucleotide sequences, and there were a total of 1020 positions in the final dataset.

Pairwise comparison of *I.lanigeri* sp. nov. indicated 5.1% *cox*1 and 2.9% 16S rRNA gene sequence differences from *I.fujitai*, and 11.18% *cox*1 and 5.7% 16S rRNA gene sequence difference from *I.ariadnae* (Table 1). There were up to only 2 bp differences in the amplified part of the *cox*1 and 16S rRNA genes between the larvae, the nymph and female of *I.lanigeri* sp. nov. The complete mitogenome of *I.lanigeri* sp. nov. was 95.4% (13899/14570 bp) identical to that of *I.fujitai* (LC769934). The phylogenetic relationships of the new tick species from Vietnam are shown in Fig. 9.

**Table 1.** Pairwise nucleotide differences between (a) *cox*1 and (b) 16S rRNA gene sequences of species belonging to the *Ixodesariadnae* complex, according to GenBank accession numbers. Asian and European data are indicated with light blue and grey background color, respectively.

(a) *cox*1 gene	*I.lanigeri* (PP079465: Vietnam)	*I.fujitai* (LC036330: Japan)	*I.ariadnae* (KJ490306: Hungary)
***I.lanigeri* (PP079465: Vietnam)**	–	94.9% (603/635)	88.9% (560/630)
***I.fujitai* (LC036330: Japan)**	94.9% (603/635)	–	89.7% (565/630)
***I.ariadnae* (KJ490306: Hungary)**	88.9% (560/630)	89.7% (565/630)	–
(b) 16S rRNA gene	***I.lanigeri* (PP081435: Vietnam)**	***I.fujitai* (LC036330: Japan)**	***I.ariadnae* (KJ490306: Hungary)**
***I.lanigeri* (PP081435: Vietnam)**	–	97.1% (398/410)	94.3% (398/422)
***I.fujitai* (LC036330: Japan)**	97.1% (398/410)	–	93% (385/414)
***I.ariadnae* (KJ490306: Hungary)**	94.3% (398/422)	93% (385/414)	–

#### Host records and distribution.

Known host species: *Myotisalticraniatus*, *M.laniger*. Known geographical range: northern Vietnam.

#### Etymology.

The name of the new species refers to the host species, the Chinese water myotis (*M.laniger*) from which the first specimen of the new species was collected.

## ﻿Discussion

Bat-associated ixodid ticks are considered to belong to three species complexes: the *I.simplex* group characterized by the normal length of legs, and two complexes of the so-called long-legged bat tick species, *I.vespertilionis* and *I.ariadnae* (Hornok et al. 2014; Hornok et al. 2015; Takano et al. 2023). Although until the discovery of the latter species, only one long-legged bat-associated ixodid tick species was known in the Old World, later these were recognized to probably represent at least six species in Eurasia, as suspected on a molecular-phylogenetic basis (Hornok et al. 2015). This was later confirmed by the description of new long-legged bat tick species in Vietnam (*Ixodescollaris*: Hornok et al. 2016b) and in Japan (*Ixodesnipponrhinolophi* and *I.fujitai*: Takano et al. 2023). Thus, with the description of *I.lanigeri* sp. nov. in this study from Vietnam, the list and description of six species which were expected to exist among “long-legged bat ticks” became complete.

Hitherto, *I.lanigeri* was probably misidentified as *I.vespertilionis* in Southeast Asia. Among bat-associated ticks of the subgenus Pholeoixodes (formerly *Escathocephalus*: Hornok et al. 2017b), *I.lanigeri* belongs to the phylogenetic group of the *I.ariadnae* complex (Takano et al. 2023) and is most closely related to *I.fujitai* with East Asian occurrence, and also to *I.ariadnae* from Europe and Asia Minor (Hekimoglu et al. 2022). For these reasons, the differential diagnosis focused on the latter two species. Morphological comparisons in this context fully supported the taxonomic status of *I.lanigeri* sp. nov. as a separate species.

*Myotis*-associated ticks collected previously in Vietnam belonged to the *I.ariadnae* complex and were thus called *I.ariadnae*-like (Hornok et al. 2015). Based on two mitochondrial genetic markers, the clustering of *I.ariadnae*-like species from Vietnam, separately from *I.ariadnae* and *I.fujitai* was moderately (77–83%) or highly (94–100%) supported, respectively (Hornok et al. 2015; Takano et al. 2023). This was confirmed here, i.e. the clustering of *I.lanigeri* sp. nov. and *I.fujitai* received high (100%) support (Fig. 9). Interestingly, the phylogenetic position and evolutionary distance of a tick reported from southern China (MW411447: Lu et al. 2021) suggest that further, taxonomically undescribed, species of the *I.vespertilionis* complex might exist in southeastern Asia (Fig. 9).

In the present study, pairwise comparisons indicated 5.1% *cox*1 and 2.9% 16S rRNA gene sequence differences from the closest related species, *I.fujitai*. Although this is lower than the average sequence divergence between closely related species (6.1% and 5.2%, respectively: Lv et al. 2014), other well-established ixodid species are known with even lower degrees of interspecific differences (e.g., *Ixodesturdus* Nakatsudi and *Ixodesfrontalis* (Panzer) differing by only 2.7% in their 16S rRNA gene sequences: Xu et al. 2003).

At the same time, there are shortcomings which originate from the rarity of *Myotis*-associated bat ticks in southeastern Asia, i.e., that the description of the new species is based on morphological analyses of a single individual of each developmental stage, and not on a representative number of specimens from a population. Considering the genetic differences between *I.lanigeri* sp. nov. and a previously reported specimen from *Myotis* sp. in Vietnam (KR902767, KR902770: Hornok et al. 2015) it is possible to assume that further (perhaps sibling) species of the *I.ariadnae* complex might also exist in this geographical region, and it will be necessary to address this in future studies.

Ixodid ticks associated with bats infrequently occur beyond the range of their typical hosts; thus, host spectra of these tick species reflect family-level adaptations. The typical hosts of the *I.vespertilionis* and *I.simplex* groups are bat species from the families Rhinolophidae and Miniopteridae, respectively (Hornok et al. 2015). Regarding the third group of bat-associated tick species, the *I.ariadnae* complex, they most frequently occur on vesper bats (family Vespertilionidae): *I.ariadnae* is the most common on *Myotis* species (Hornok et al. 2014), and *I.lanigeri* sp. nov. was only reported so far from bats of this genus, whereas *I.fujitai* only from *Murinahilgendorfi* (Takano et al. 2023). It is important to note that although both genera, *Myotis* and *Murina*, belong to Vespertilionidae, they are not sister-genera and are generally placed into two distinct subfamilies, Myotinae and Murininae (Jargalsaikhan et al. 2022). Apart from their distinct geographical range (see below), this may have contributed to, and may in part explain, the speciation events that most likely resulted in the divergent evolution of *I.lanigeri* sp. nov. and *I.fujitai*.

Among bat species of genera *Myotis* and *Murina*, the typical hosts of *I.lanigeri* sp. nov. and *I.fujitai* are geographically separated between south and southeastern and East Asia (Fig. 1). In particular, the majority of *Myotis* and *Murina* species indigenous in Vietnam (i.e., the expected hosts of *I.lanigeri* sp. nov.) have a south-southeastern Asian distribution: their geographical range including the Himalayan and Indian subregions (*N* = 10 bat species), the Indochinese (*N* = 35 bat species) subregion, as well as the Sundaic, Wallacean and Philippine (*N* = 7 bat species) subregions. However, only two of the Vietnamese *Myotis* species occur in Palearctic East Asia, and none of them on the five main islands of Japan where *I.fujitai* was reported from its type host, *Mu.hilgendorfi* (Fig. 1).

On the other hand, it has to be noted that the type host of *I.lanigeri* sp. nov., *My.lanigeri* is known to roost in sympatry with *My.fimbriatus* and *My.altarium* (Hu et al. 2012); therefore, the new tick species almost certainly has a larger geographical range, including China and other countries in south-southeastern Asia, where these bat species occur. This was reflected by the clustering of a long-legged bat tick species (reported recently from Eastern China: Tian et al. 2022) with *I.lanigeri* sp. nov. (Fig. 9).

By contrast, typical bat host species (Miniopteridae) of the *I.simplex* group show considerable overlapping in their geographical distribution in East and Southeast Asia. For example, the most important host species of *I.fuliginosus* described recently in Japan (i.e., *Miniopterusfuliginosus*) also occurs in Vietnam, and *Miniopterusmagnater* from which a phylogenetically divergent genetic variant of *I.simplex* was reported from India (Hornok et al. 2015) is also found in Vietnam (Kusuminda et al. 2022). Therefore, due to this connectedness and in the absence of clear geographical separation, it is likely that so-called “short-legged bat ticks” of the *I.simplex* group in South, Southeast and East Asia probably belong to the same species, *I.fuliginosus*.

## Supplementary Material

XML Treatment for Ixodes lanigeri

## ﻿Additional information

### Author ORCIDs

Sándor Hornok https://orcid.org/0000-0002-1125-5178

Jenő Kontschán https://orcid.org/0000-0001-8274-4238

Ai Takano https://orcid.org/0000-0002-5919-3738

Alexandre Hassanin https://orcid.org/0000-0002-4905-8540

Vuong Tan Tu https://orcid.org/0000-0002-5915-865X

### Data availability

All of the data that support the findings of this study are available in the main text or Supplementary Information.

## Supplementary materials

Supplementary material 1 Simplified geographical range of *Myotis* and *Murina* species Data type pdf Explanation note Simplified geographical range of *Myotis* and *Murina* species (Chiroptera: Vespertilionidae) found in Vietnam and Laos (based on IUCN (2023), Wilson & Mittermeier (2019) and selected references listed below). Species found only in Vietnam are indicated with red fonts. Known hosts of the new bat tick species are indicated with yellow background. https://binary.pensoft.net/file/1153133This dataset is made available under the Open Database License (http://opendatacommons.org/licenses/odbl/1.0/). The Open Database License (ODbL) is a license agreement intended to allow users to freely share, modify, and use this Dataset while maintaining this same freedom for others, provided that the original source and author(s) are credited.Sándor Hornok, Vuong Tan Tu
