# Supplementary material for: ﻿Description of Ixodeslanigeri sp. nov., a new hard tick species (Acari, Ixodidae) collected from mouse-eared bats (Vespertilionidae, Myotis) in Vietnam
Source: Zookeys. 2024 Oct 14;1215:107–25. doi: 10.3897/zookeys.1215.123624 (PMC11494211; doi:10.3897/zookeys.1215.123624)
Supplement: Supplementary material 1 — Simplified geographical range of Myotis and Murina species [file zookeys-1215-107_article-123624__-s001.pdf]

Supplementary Table 1. Simplified geographical range of *Myotis* and *Murina* species (Chiroptera: Vespertilionidae) found in Vietnam and Laos (based on IUCN (2023), Wilson & Mittermeier (2019) and selected references listed below). Species found only in Vietnam are indicated with red fonts. Known hosts of the new bat tick species are indicated with yellow background.

#	Taxon	Indomalayan subregions																								Palearctic region (East Asia)		
		Indochinese								Sundaic					Indian					Himalayan			Wallacean		Philippine			
		Vietnam	Laos	Cambodia	S. China	Taiwan	Thailand	Myanmar	And-Nic Is.	Malaysia	Sumatra	Singapore	Boneo	Java	Sri Lanka	S. India	Pakistan	Banglades	Afghanistan	Bhutan	Nepal	NE. India	Sulawesi	Maluku	Philippines	Japan	Korea	NE. China
1	*My. altarium* Thomas, 1911	+			+		+															+						
2	*My. ancricola* Kruskop et al., 2018	+	+																									
3	*My. annatessae* Kruskop & Borisenko, 2013	+	+																									
4	*My. annamiticus* Kruskop & Tsytsulina, 2001	+	+																									
5	*My. annectans* (Dobson, 1871)	+	+	+	+		+															+						
6	*My. ater* (Peters, 1866)	+		+			+			+			+	+									+	+	+			
7	*My. chinensis* (Tomes, 1857)	+			+		+	+																				
8	*My. hasseltii* (Temminck, 1840)	+	?	+	+		+	+		+	+		+	+	+	+												
9	*My. horsfieldii* (Temminck, 1840)	+	+	+	+		+	+	+	+		+	+	+									+		+			
10	*My. indochinensis* Son et al., 2013	+	+		+																							
11	*My. laniger* (Peter, 1871)	+			+																	+						
12	*My. montivagus* (Dobson, 1874)	+	+		+			+																				
13	*My. muricola* (Gray, 1846)	+	+	+	+		+	+		+	+	+	+	+		+				+	+	+	+	+	+			
14	*My. phanluongi* Borisenko et al. 2008	+																										
15	*My. pilosus* (Peters, 1869)	+	+		+																							+
16	*My. rosseti* (Oey, 1951)	+	+	+			+																					
17	*My. siligorensis* (Horsfield, 1855)*	+	+	+	+		+	+		+			+															
18	*My. formosus* (Hodgson, 1835)	+			+	+										+	+		+	+	+	+						
19	*My. rufoniger* Tomes 1858	+	+		+	+																				TI	+	+
20	*Harpiocephalus harpia* (Temminck, 1840)	+	+	+	+	+	+	+		+	+		+	+		+						+			+			
21	*Harpiola isodon* Kuo et al., 2006	+				+																						
22	*Mu. annamitica* Francis & Eger, 2012	+	+				+																					
23	*Mu. beelzebub* Son et al., 2011	+																										

#	Taxon	Indomalayan subregions																								Palearctic region (East Asia)		
		Indochinese								Sundaic					Indian					Himalayan			Wallacean		Philippine			
		Vietnam	Laos	Cambodia	S. China	Taiwan	Thailand	Myanmar	And-Nic Is.	Malaysia	Sumatra	Singapore	Boneo	Java	Sri Lanka	S. India	Pakistan	Banglades	Afghanistan	Bhutan	Nepal	NE. India	Sulawesi	Maluku	Philippines	Japan	Korea	NE. China
24	*Mu. chrysochaetes* Eger & Lim, 2011	+			+																							
25	*Mu. cyclotis* Dobson, 1872	+	+	+	+		+	+							+	+					+	+			+			
26	*Mu. eleryi* Furey et al., 2009	+	+		+																							
27	*Mu. feae* (Thomas, 1891)	+	+	+	+		+	+																				
28	*Mu. fionae* Francis & Eger, 2012	+	+	+																								
29	*Mu. harpioloides* Kruskop & Eger, 2008	+																										
30	*Mu. harrisoni* Crosba & Bates, 2005	+	+	+	+		+	+																				
31	*Mu. huttoni* (Peters, 1872)	+	+		+		+	+		+											+	+						
32	*Mu. kontumensis* Son et al., 2015	+																										
33	*Mu. leucogaster* Milne-Edwards, 1872	+			+																	+						
34	*Mu. lorelieae* Eger & Lim, 2011	+			+																							
35	*Mu. walstoni* Furey et al., 2011	+	+	+																								

Abbreviations: S – South; NE – Northeast; And-Nic Is.- Andaman and Nicobar Islands; TI – only Tsushima Island; ? – uncertain
Taxonomic notes: * - According to Ruedi et al. (2021), *Myotis siligorensis* is endemic to Himalayan subregion, while bats formerly identified as this taxon found in Indochinese subregion should be *My. alticraniatus* Osgood, 1932

References

IUCN (2023). The IUCN Red List of Threatened Species. Version 2022–2. Retrieved from http://www.iucnredlist.org.

Kruskop SV, Borisenko AV, Dudorova AV, Artyushin IV (2018) Description of a new Indochinese Myotis (Mammalia: Chiroptera: Vespertilionidae), with additional data on the "M. annatessae" species complex. Russian Journal of Theriology 17: 17–31.

Ruedi M, Saikia U, Thabah A, Görföl T, Thapa S, Csorba G (2021) Molecular and morphological revision of small Myotinae from the Himalayas shed new light on the poorly known genus Submyotodon (Chiroptera: Vespertilionidae). Mammalian Biology 101: 465–480.

Wilson DE, Mittermeier RA (Eds) (2019) Handbook of the Mammals of the World. Lynx Edicions, Barcelona.
